# Supplementary material for: Nested Markov chain hyper-heuristic (NMHH): a hybrid hyper-heuristic framework for single-objective continuous problems
Source: PeerJ Comput Sci. 2024 Feb 2;10:e1785. doi: 10.7717/peerj-cs.1785 (PMC10909227; doi:10.7717/peerj-cs.1785)
Supplement: Supplemental Information 1 [file peerj-cs-10-1785-s001.pdf]

Table S1. Results of parameter tuning on the Rosenbrock function in 50, 100 and 500 dimensions

Dim.	Function eval.	Min median + IQR	SA steps	SA temp	SA alpha
50	100	5.698500e+01	50	1000	5.0
50	100	4.583468e+01	100	1000	5.0
50	100	4.569869e+01	200	1000	5.0
50	100	4.661390e+01	50	10000	5.0
50	100	4.550024e+01	100	10000	5.0
50	100	4.650158e+01	200	10000	5.0
50	100	4.615916e+01	50	1000	50.0
50	100	**4.537342e+01**	100	**1000**	50.0
50	100	4.552422e+01	200	1000	50.0
50	100	2.678746e+03	50	10000	50.0
50	100	4.619361e+01	100	10000	50.0
50	100	4.576032e+01	200	10000	50.0
50	5000	4.427826e-11	50	1000	5.0
50	5000	3.689173e-21	100	1000	5.0
50	5000	**0.000000e+00**	200	**1000**	5.0
50	5000	8.668238e-13	50	10000	5.0
50	5000	**0.000000e+00**	100	**10000**	5.0
50	5000	**0.000000e+00**	200	**10000**	5.0
50	5000	6.781357e-11	50	1000	50.0
50	5000	1.672572e-11	100	1000	50.0
50	5000	1.236658e-14	200	1000	50.0
50	5000	**0.000000e+00**	50	**10000**	50.0
50	5000	4.509591e-12	100	10000	50.0
50	5000	**0.000000e+00**	200	**10000**	50.0
100	100	8.574862e+03	50	1000	5.0
100	100	9.588105e+01	100	1000	5.0
100	100	1.518986e+03	200	1000	5.0
100	100	9.573063e+01	50	10000	5.0
100	100	9.594477e+01	100	10000	5.0
100	100	**9.466789e+01**	200	**10000**	5.0
100	100	9.591726e+01	50	1000	50.0
100	100	9.530534e+01	100	1000	50.0
100	100	9.494694e+01	200	1000	50.0
100	100	9.697318e+01	50	10000	50.0
100	100	9.620821e+01	100	10000	50.0
100	100	9.535291e+01	200	10000	50.0
100	5000	3.483557e+01	50	1000	5.0
100	5000	5.461610e+00	100	1000	5.0
100	5000	3.384631e+01	200	1000	5.0
100	5000	3.830000e+01	50	10000	5.0
100	5000	3.292423e+01	100	10000	5.0
100	5000	3.310204e+01	200	10000	5.0
100	5000	3.343096e+01	50	1000	50.0
100	5000	3.317775e+01	100	1000	50.0
100	5000	3.394381e+01	200	1000	50.0
100	5000	3.434196e+01	50	10000	50.0
100	5000	3.112265e+01	100	10000	50.0
100	5000	**0.000000e+00**	200	**10000**	50.0
500	100	4.917224e+02	50	1000	5.0
500	100	5.901930e+04	100	1000	5.0
500	100	4.916484e+02	200	1000	5.0
500	100	4.935340e+02	50	10000	5.0
500	100	**4.909480e+02**	100	**10000**	5.0
500	100	4.917368e+02	200	10000	5.0
500	100	4.932843e+02	50	1000	50.0
500	100	5.160497e+04	100	1000	50.0
500	100	4.916740e+02	200	1000	50.0
500	100	3.128190e+04	50	10000	50.0
500	100	4.928036e+02	100	10000	50.0
500	100	4.917936e+02	200	10000	50.0
500	5000	4.329341e+02	50	1000	5.0
500	5000	**2.993221e+02**	100	**1000**	5.0
500	5000	4.307063e+02	200	1000	5.0
500	5000	4.321560e+02	50	10000	5.0
500	5000	4.332272e+02	100	10000	5.0
500	5000	3.924223e+02	200	10000	5.0
500	5000	4.307235e+02	50	1000	50.0
500	5000	4.333202e+02	100	1000	50.0
500	5000	4.291124e+02	200	1000	50.0
500	5000	4.353929e+02	50	10000	50.0
500	5000	4.334789e+02	100	10000	50.0
500	5000	3.079623e+02	200	10000	50.0
